# Supplementary material for: Large-area, periodic, and tunable intrinsic pseudo-magnetic fields in low-angle twisted bilayer graphene
Source: Nat Commun. 2020 Jan 17;11:371. doi: 10.1038/s41467-019-14207-w (PMC6969151; doi:10.1038/s41467-019-14207-w)
Supplement: Supplementary file 1 — Supplementary Information [file 41467_2019_14207_MOESM1_ESM.pdf]

Supplementary Information

Large-area, periodic, and tunable intrinsic pseudo-magnetic fields in low-angle twisted bilayer graphene

Shi et al.

Large-area, periodic, and tunable intrinsic pseudo-magnetic fields in low-angle twisted bilayer graphene: Supplementary Information

Haohao Shi,[1,2,*] Zhen Zhan,[3,*] Zhikai Qi,[4] Kaixiang Huang,[3] Edo van Veen,[5] Jose Ángel Silva-Guillén,[3] Runxiao Zhang,[1,2] Pengju Li,[1,2] Kun Xie,[1,2] Hengxing Ji,[4] Mikhail I. Katsnelson,[5] Shengjun Yuan,[3,†] Shengyong Qin,[1,2,‡] and Zhenyu Zhang[1]

[1]*International Centre for Quantum Design of Functional Materials (ICQD), Hefei National Laboratory for Physical Sciences at the Microscale (HFNL), and Synergetic Innovation Center of Quantum Information and Quantum Physics, University of Science and Technology of China, Hefei, 230026, China*

[2]*CAS Key Laboratory of Strongly-Coupled Quantum Matter Physics, Department of Physics, University of Science and Technology of China, Hefei, 230026, China*

[3]*Key Laboratory of Artificial Micro- and Nano-structures of Ministry of Education and School of Physics and Technology, Wuhan University, Wuhan 430072, China*

[4]*Hefei National Laboratory for Physical Sciences at the Microscale, Department of Applied Chemistry, CAS Key Laboratory of Materials for Energy Conversion, iChEM (Collaborative Innovation Center of Chemistry for Energy Materials), University of Science and Technology of China, Hefei, 230026, China*

[5]*Radboud University, Institute for Molecules and Materials, NL-6525 AJ Nijmegen, The Netherlands*

(Dated: December 10, 2019)

Supplementary Note 1. SEM and Raman results

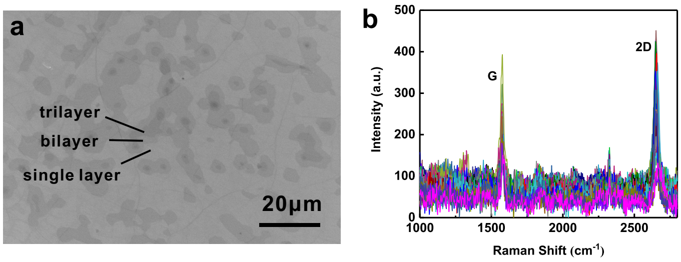

Supplementary Figure 1. **SEM image and Raman spectra of bilayer graphene on Cu-Ni substrate.** **a**, Typical large area SEM image of the bilayer graphene sample grown on Cu-Ni substrate. The bilayer domains can be clearly visualized with high coverages. **b**, 45 Raman spectra taken on the same area in **a** with average distance of 10 μm.

The SEM and Raman spectra of bilayer graphene are plotted in Supplementary Fig. 1. In the SEM image, we can easily identify the single layer, bilayer and even trilayer graphene sample with different coverages. Due to the growth mechanism in Cu-Ni substrate, the fully covered single layer graphene (first layer) is at top most and the second layer is right under the first one and so on. The small area and low density of trilayer islands makes it hard to be located in our STM scanning images while the bilayers can be positioned with appearance of various moiré patterns. For the Raman measurements, the position of G peak is at 1587.9 cm^{-1} and the 2D peak at 2711.3 cm^{-1}. In the all 45 cases, the intensity of G and 2D ($I_{2D}/I_G < 2$) peaks and absent D peak indicates high quality bilayer graphene character. The SEM measurements were conducted with a field emission scanning electron microscopy (JSM-6700F, JEOL Ltd.) operated at 10.0 kV. Raman spectroscopy of graphene samples was characterized by LABRAM-HR with a 532nm laser.

Supplementary Note 2. Angle dependence of van Hove singularities

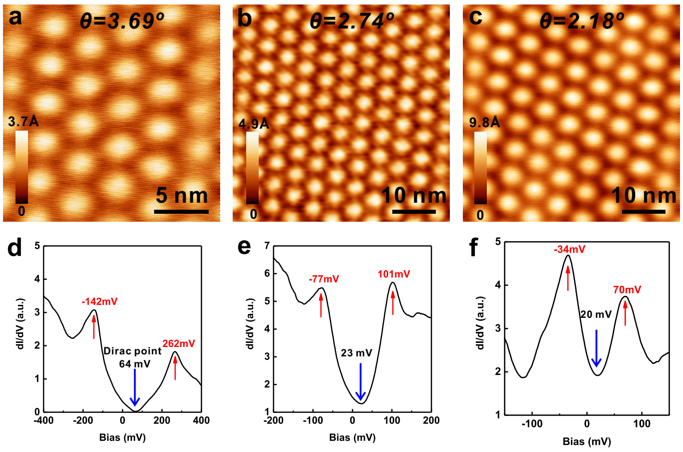

Supplementary Figure 2. **STM images and dI/dV spectra with different twist angles.** **a** and **d**, Twist angle $\theta = 3.69°$ with $\Delta_{\mathrm{VHS}} = 304$ mV. **b** and **e**, Twist angle $\theta = 2.74°$ with $\Delta_{\mathrm{VHS}} = 178$ mV. **c** and **f**, Twist angle $\theta = 2.18°$ with $\Delta_{\mathrm{VHS}} = 104$ mV.

In this section, the measured dI/dV conductance for TBG with twist angles larger than 2° are presented in Supplementary Fig. 2. In the STM images, all the moiré patterns show nearly perfect sixfold symmetry, indicating here that the strain effects on its electronic properties can be neglected. The tendency of the two low energy van

Hove singularities for TBG with different twist angle are highly consistent with previous calculated results in Ref. 1. Moreover, since all the twist angle are larger than the crossover angle $\theta^\star = 1.2°$, the structural deformation has negligible effects on the local density of states [1].

Supplementary Note 3. Background subtraction fittings of dI/dV spectra of twist angle $\theta = 0.48°$

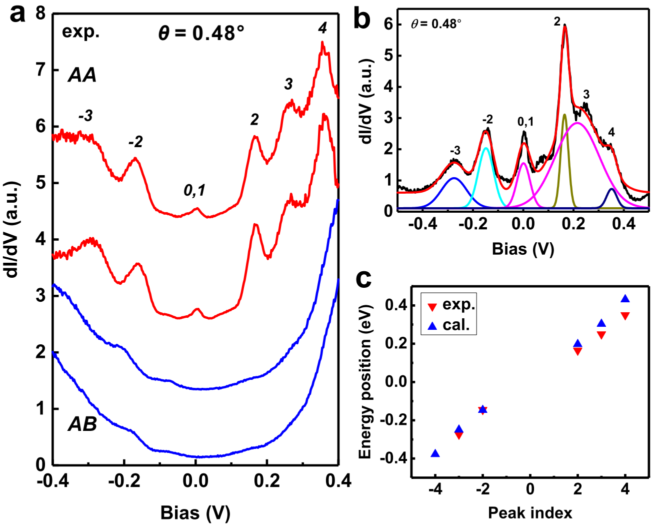

Supplementary Figure 3. **Multiple peaks Gaussian fittings of the dI/dV spectra with twist angle $\theta = 0.48°$.** **a**, Representative dI/dV spectra taken at the separate locations of AA and AB regions, which is consistent with the logarithmic spectra displayed in Fig. 1 of the main text. **b**, Background subtracted sepctra in Supplementary Fig. 3a. **c**, Comparison of the experimental and theoretical results of the pseudo-Landau levels.

To obtain the more distinct pseudo-Landau level peaks as displayed in Fig. 1b, the raw dI/dV data (Supplementary Fig. 3a) was subtracted with background and then fitted by multiple Gaussian fitting analysis. The results are plotted in Supplementary Fig. 3b where the pseudo-Landau levels are labeled as integer numbers. We also plot the calculated and experimental results obtained from Fig. 1 of the main text as displayed in Supplementary Fig. 3c, which are in good agreement.

Supplementary Note 4.Graphene layer pinning at the grain boundary

The strained region forms at the grain boundaries of the bilayer graphene or step edges of the CuNi alloyed surface in the epitaxial growth stage as shown in Supplementary Fig. 4a. Here, the top layer graphene is unstrained and the bottom layer is slightly strained along one direction as we can derive from the STM image (that

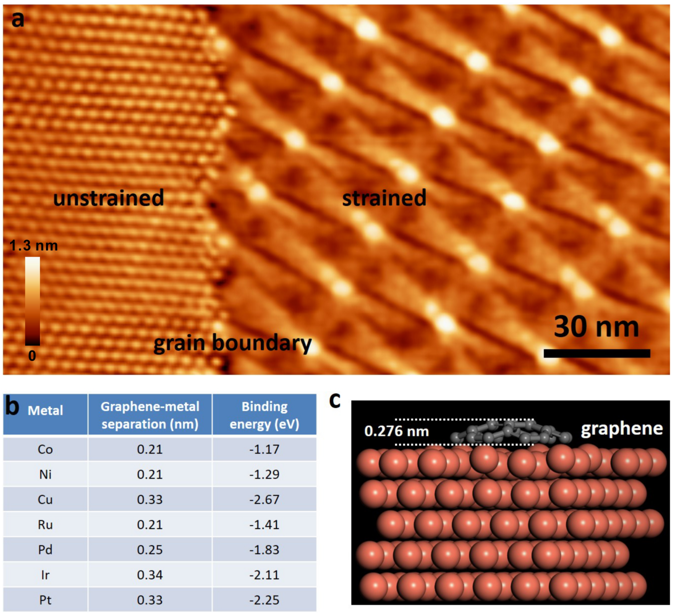

Metal	Graphene-metal separation (nm)	Binding energy (eV)
Co	0.21	-1.17
Ni	0.21	-1.29
Cu	0.33	-2.67
Ru	0.21	-1.41
Pd	0.25	-1.83
Ir	0.34	-2.11
Pt	0.33	-2.25

Supplementary Figure 4. **Origin of the uniaxial heterostrain.** **a**, A representive topography image of unstriained and strained regions of twisted bilayer graphene as well as the grain boundary at their junction. **b**, The interlayer couplings of graphene/metal interface [4]. **c**, Calculated results of the relaxed graphene island on Cu(111) surface.

is, the deformed moiré pattern) and our atomic simulations. The strained region with deformed moiré pattern in the right part meets with the unstrained region, where the grain boundary can be clearly observed. This result agrees well with previous studies of the origin of the heterostrain in the low-angle TBG [2].

Earlier theoretical studies of graphene/metal interface show that the separation distance is directly related to the changes in the electronic structure of graphene [3]. An absolute binding energy less than 2 eV can be considered as weak interaction at the graphene/metal interface [4]. Therefore, for the Cu atom dominated CuNi(111) surface, it is logical to deduce that the alloyed surface is weakly bonded with the graphene layers and the pseudo-magnetic field behavior is not originate from the substrates. We have also performed density functional theory calculations by relaxing the graphene islands on the Cu(111) surface as shown in Supplementary Fig 4c. The density functional theory calculations are performed by using the Vienna ab initio simualtion package (VASP) with PAW potentials and generalized gradient approximation (PBE-GGA) for the exchange-correlation functional [5]. We find that the graphene has a dome-like structure, where the central carbon atoms are 0.276 nm from the surface. The interlayer interactions are rather weak between the central C atoms and the substrate. However, the C atoms at the periphery are strongly bonded with the metal atoms, forming a sp3-like hy-

bridizations. Such results are highly consistent with earlier reports [5]. Thus, we suggest that strongly bonded graphene edge (that is, the grain boundary) provides an extra driving force to form uniaxial heterostrain as has been clarified above.

Supplementary Note 5. Geometry of twisted and strained bilayer graphene

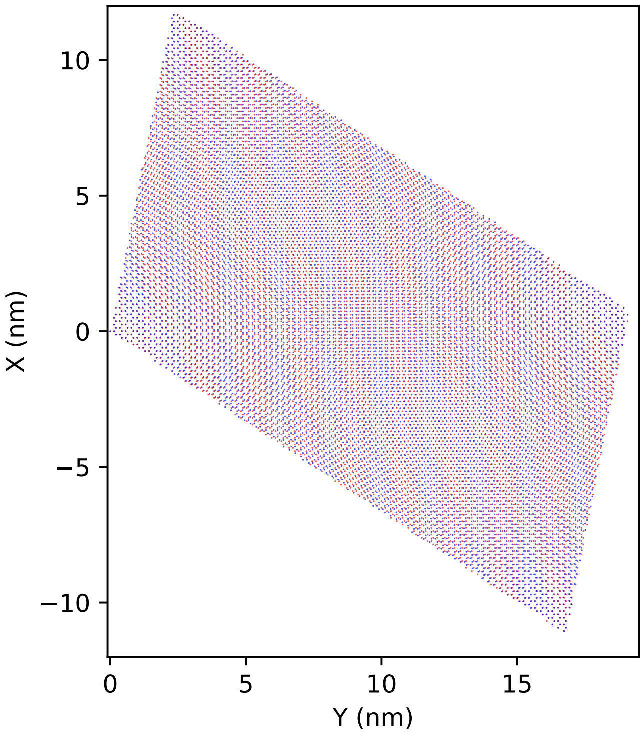

Supplementary Figure 5. **Moiré cell.** One supercell of twisted bilayer graphene with twist angle $\theta = 0.98^\circ$ and heterostrain $\sigma = 0.78\%$. Blue points: layer with strain, red points: layer without strain.

Generally, for twisted bilayer graphene without strain, the supercell can be constructed by identifying a common periodicity between the two layers, and the size of the moiré pattern is $a_m = \sqrt{3}d/[2|\sin(\theta/2)|]$, being d the carbon-carbon distance.

We define homostrain as the case where both layers suffer the same strain and heterostrain as that where the two layers have a different strain. In a more general case where, for instance, graphene is twisted, strained and sheared with respect to the substrate, the commensurate relation between graphene and the substrate can be explicitly described by a set of eight integers (i, j, k, l, m, n, q, r). Let us assume that the lattice vectors of graphene are $(\boldsymbol{a_{o1}}, \boldsymbol{a_{o2}})$, which form an angle ω. The lattice vectors of the substrate $(\boldsymbol{a'_{o1}}, \boldsymbol{a'_{o2}})$ form an angle ω'. Then, the lattice vectors of the superstructure are:

$$\begin{pmatrix} \boldsymbol{a_{m1}} \\ \boldsymbol{a_{m2}} \end{pmatrix} = \begin{pmatrix} i & j \\ k & l \end{pmatrix} \begin{pmatrix} \boldsymbol{a_{o1}} \\ \boldsymbol{a_{o2}} \end{pmatrix} = \begin{pmatrix} m & n \\ q & r \end{pmatrix} \begin{pmatrix} \boldsymbol{a'_{o1}} \\ \boldsymbol{a'_{o2}} \end{pmatrix}. \quad (1)$$

The lengths of the moiré pattern are:

$$\begin{aligned} |\boldsymbol{a_{m1}}| &= \sqrt{(i\boldsymbol{a_{o1}})^2 + (j\boldsymbol{a_{o2}})^2} = \sqrt{(m\boldsymbol{a'_{o1}})^2 + (n\boldsymbol{a'_{o2}})^2}, \\ |\boldsymbol{a_{m2}}| &= \sqrt{(k\boldsymbol{a_{o1}})^2 + (l\boldsymbol{a_{o2}})^2} = \sqrt{(q\boldsymbol{a'_{o1}})^2 + (r\boldsymbol{a'_{o2}})^2}. \end{aligned} \quad (2)$$

The general form of the Park-Madden matrix can be written as [6]:

$$\begin{aligned} \begin{pmatrix} \boldsymbol{a_{o1}} \\ \boldsymbol{a_{o2}} \end{pmatrix} &= \begin{pmatrix} A & B \\ C & D \end{pmatrix} \begin{pmatrix} \boldsymbol{a'_{o1}} \\ \boldsymbol{a'_{o2}} \end{pmatrix} \\ &= \frac{1}{il - jk} \begin{pmatrix} lm - jq & ln - jr \\ -km + iq & -kn + ir \end{pmatrix} \begin{pmatrix} \boldsymbol{a'_{o1}} \\ \boldsymbol{a'_{o2}} \end{pmatrix}. \end{aligned} \quad (3)$$

The number of beatings N in a moiré cell is:

$$\begin{aligned} N = &\sqrt{(i-m)^2 + (j-n)^2 - (i-m)(j-n)} \\ &\times \sqrt{(k-q)^2 + (l-r)^2 - (k-q)(l-r)}. \end{aligned} \quad (4)$$

On the other hand, the (2×2) matrix can be rewritten with the extended Wood's notation $(P_1\boldsymbol{R}\theta_1, P_2\boldsymbol{R}\theta_2)$ [7]:

$$\begin{pmatrix} \boldsymbol{a_{o1}} \\ \boldsymbol{a_{o2}} \end{pmatrix} = \frac{1}{\sin\omega} \begin{pmatrix} P_1 \sin(\omega - \theta_1) & \frac{|\boldsymbol{a_{o1}}|}{|\boldsymbol{a_{o2}}|} P_1 \sin\theta_1 \\ -\frac{|\boldsymbol{a_{o2}}|}{|\boldsymbol{a_{o1}}|} P_2 \sin\theta_2 & P_2 \sin(\omega' + \theta_2) \end{pmatrix} \begin{pmatrix} \boldsymbol{a'_{o1}} \\ \boldsymbol{a'_{o2}} \end{pmatrix}, \quad (5)$$

where the factors $P_1 = |\boldsymbol{a_{o1}}|/|\boldsymbol{a'_{o1}}|$ and $P_2 = |\boldsymbol{a_{o2}}|/|\boldsymbol{a'_{o2}}|$, the rotated angles $\theta_1 = (\boldsymbol{a_{o1}}, \boldsymbol{a'_{o1}})$, $\theta_2 = (\boldsymbol{a_{o2}}, \boldsymbol{a'_{o2}})$. For twisted bilayer graphene with $\omega = \omega' = 120^\circ$, Supplementary Eq. (5) becomes:

$$\begin{pmatrix} \boldsymbol{a_{o1}} \\ \boldsymbol{a_{o2}} \end{pmatrix} = \begin{pmatrix} P_1\left(\cos\theta_1 + \frac{\sin\theta_1}{\sqrt{3}}\right) & \frac{2P_1}{\sqrt{3}} \sin\theta_1 \\ -\frac{2P_2}{\sqrt{3}} \sin\theta_2 & P_2\left(\cos\theta_2 - \frac{\theta_2}{\sqrt{3}}\right) \end{pmatrix} \begin{pmatrix} \boldsymbol{a'_{o1}} \\ \boldsymbol{a'_{o2}} \end{pmatrix}. \quad (6)$$

Supplementary Eqs. (3) and (6) are identical, therefore, we can write the parameters P_1, P_2, θ_1 and θ_2 with the eight integers, as

$$\begin{aligned} P_1 &= \sqrt{A^2 + B^2 - AB}, \\ P_2 &= \sqrt{C^2 + D^2 - CD}, \\ \theta_1 &= \arctan\left(\frac{B\sqrt{3}}{2A - B}\right), \\ \theta_2 &= \arctan\left(\frac{C\sqrt{3}}{C - 2D}\right). \end{aligned} \quad (7)$$

For a TBG with given moiré wavelengths in the real space, we numerically solve the Supplementary Eq. (3) to obtain a unique set of eight integers. Then, using the formula in Supplementary Eq. (7),

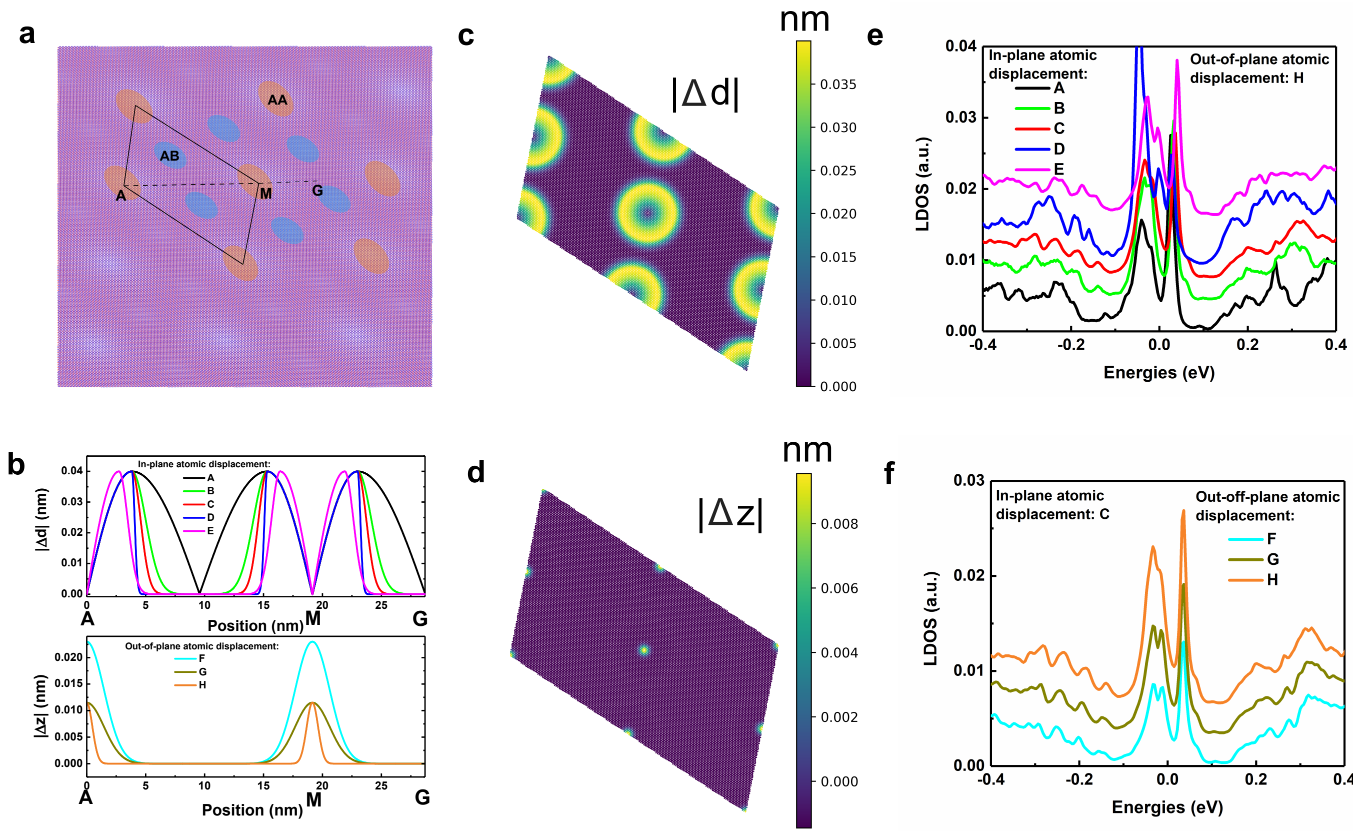

Supplementary Figure 6. **Relaxed atomic structure of twisted bilayer graphene characterized by $\theta = 0.98^\circ$ and $\sigma = 0.78\%$.** **a**, Schematic model of the moiré pattern. **b**, Absolute magnitude of different in-plane atomic displacements (top panel) and out-of-plane displacements for the relaxed system along the path AMG defined in **a**. For the in-plane displacement, the parameters (ΔD_{max}, L_D, σ_D) are: A (0.04 nm, 3.83 nm, 1089.9), B (0.04 nm, 3.83 nm, 2.72), C (0.04 nm, 3.83nm, 1.09), D (0.04 nm, 3.83 nm, 0.11), E (0.04 nm, 2.74 nm, 1.09). For the out-of-plane displacements, the parameters (ΔZ_{max}, L_Z, σ_Z) are: F (0.023 nm, 6.39 nm, 4.26), G (0.0115 nm, 6.39 nm, 4.26), H (0.0115 nm, 3.19 nm, 0.46). **c** and **d**, Maps of the absolute magnitude of the in-plane atomic displacement $|\Delta d|$ and out-of-plane displacement Δz upon deformation of rigidly TBG, respectively. LDOS in the AA region of relaxed systems with different in-plane displacements in **e** and different out-of-plane displacements in **f**.

we can obtain the four unknown parameters P_1, P_2, θ_1 and θ_2 that best fit to the three measured moiré wavelengths. For example, for the Fig. 1 in the main text, the eight integers are $(i, j, k, l, m, n, q, r) = (139, 69, -69, 70, 139, 70, -70, 69)$, which give the extended Wood's notation $(1.0\boldsymbol{R}0.48, 1.0\boldsymbol{R}0.48)$. Each supercell contains one moiré patttern with the moiré length $|\boldsymbol{a_{m1}}| = |\boldsymbol{a_{m2}}| = |\boldsymbol{a_{m1}} + \boldsymbol{a_{m2}}| = 29.6$ nm and the twist angle is $\theta = 0.48^\circ$. Whereas in the TBG plotted in Fig. 4 in the main text, the three measured wavelengths are $|\boldsymbol{a_{m1}}| = 20.2$ nm, $|\boldsymbol{a_{m2}}| = 12.2$ nm and $|\boldsymbol{a_{m1}} + \boldsymbol{a_{m2}}| = 19.2$ nm, which gives the eight integers $(i, j, k, l, m, n, q, r) = (85, 78, -42, 12, 85, 79, -43, 11)$ and the extended Wood's notation $(1.0078\boldsymbol{R}1.0315, 1\boldsymbol{R}0.9818)$. As plotted in Supplementary Fig. 5, the number of beatings in the moiré cell is $N = 1$.

Supplementary Note 6. Structural deformation

In this section, we detail how we take into account the atomic relaxation in the rigidly generated moiré pattern. The relaxed system is assumed to have the same periodicity as the rigidly TBG before relaxation. It has been reported that both the in-plane and out-of-plane distortions become non-negligible and cannot be described by a sinusoidal function for TBG with twist angle $\theta < 1.2^\circ$ [8, 9]. Additionally, as shown in Fig. 4 of Ref. [9], the maximum in-plane (ΔD_{max}) and out-of-plane displacements (ΔZ_{max}) are strongly dependent on the twisted angle. Following the relaxed structures obtained from the large-scale molecular dynamics simulations for TBG with small twist angles in Refs. [8, 9], i.e., the profiles of the lattice displacements of TBG with $\theta = 0.46^\circ$ shown in Fig.4(c) and Fig.6(c) of Ref. [8] and with $\theta = 0.235^\circ$ in Fig.2 (c-d) of Ref. [9], the in-plane displacement Δd and out-of-plane displacement Δz of individual atoms can be

approximately expressed as:

$$\Delta d(x) = \Delta D_{max} \cdot \sin\left(\frac{\pi x}{2L_D}\right)\Big[1 - \Theta(x - L_D)\Big] + \Delta D_{max} \cdot \exp\Big[-\frac{(x - L_D)^2}{\sigma_D}\Big]\Theta(x - L_D),$$

$$\Delta z(x) = \pm\Delta Z_{max} \cdot \exp\Big[-\frac{x^2}{\sigma_Z}\Big]\Big[1 - \Theta(x - L_Z)\Big], \quad (8)$$

where x is the distance between the individual atom and the nearest AA point in the x-y plane, and Θ is the Heaviside step function. For the in-plane deformation, individual atoms move Δd towards the nearest AA point. In the out-of-plane deformation, the individual atom has a displacement in the z direction with $(+)$ sign for the top layer and $(-)$ sign for the bottom layer. The parameters ΔD_{max}, L_D, σ_D, ΔZ_{max}, L_Z and σ_Z determine the profile of the deformed structure and are fitting parameters to generate proper atomic structures with electronic properties matching to the experimental observations. For example, for TBG with twist angle $\theta = 0.98°$ and heterostrain $\sigma = 0.78\%$, the profiles of the atomic deformations using Supplementary Eq. (8) with different structual parameters are plotted in Supplementary Fig. 6b. The local density of states (LDOS) in the AA region of these deformed systems are also calculated. In Supplementary Fig. 6e, the LDOS changes drastically upon the in-plane relaxation. On the contrary, the out-of-plane displacements have less impact to the LDOS, as shown in Fig. 6f. As expected, the LDOS of the deformed system (with lines C and H in Supplementary Fig. 6b) have a better agreement with the measured one in Fig. 4c in the main text.

Supplementary Note 7. Pseudo-Landau levels in twisted angle $\theta = 0.65°$

According to our theory, for the strongly coupled bilayer graphene, the arising PMF increases with the decreasing twist angles due to the enhanced atomic displacements of the system. In this section, we show another low-angle TBG ($\theta = 0.65°$) as displayed in Supplementary Fig. 7. We observed the well-defined pseudo-Landau levels in Supplementary Fig.7c and the extracted PMF is about 8T as shown in Supplementary Fig. 7d.

Supplementary Note 8. The effect of heterostrain and deformation on the electronic properties with twist angle $\theta = 0.98°$

To obtain a deeper understanding of the surprising resonances in the experimental results in the main text, we have studied the effect of the heterostrain and atomic deformation on the LDOS in both AA and AB regions. Generally, as shown in Fig. 8a, for TBG with $\theta = 0.98°$

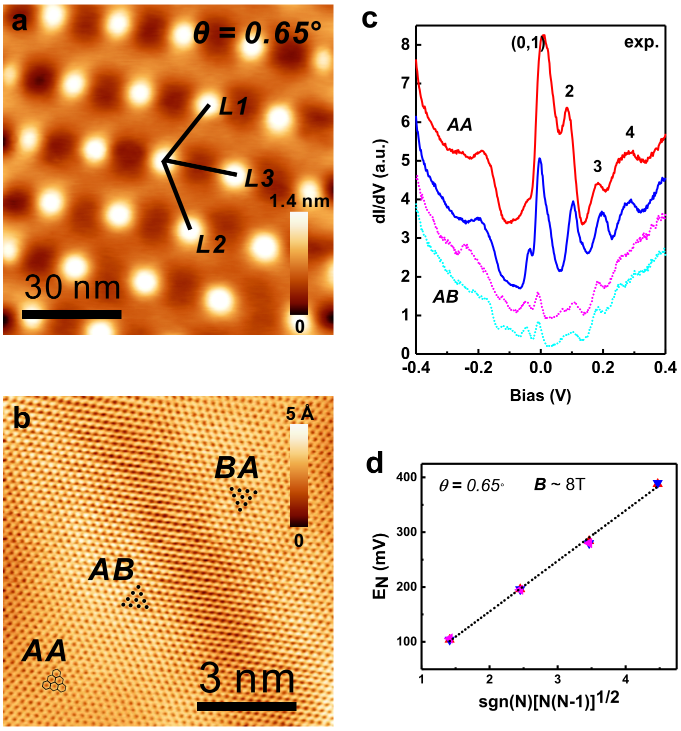

Supplementary Figure 7. **Pseudo-Lndau levels in TBG with twist angle $\theta = 0.65°$.** **a** and **b**, Topography image of 100 nm × 100 nm moiré pattern and atomic resolutions of AA, AB, and BA stacking regions. The three moiré wavelengths are: $L1 \approx L2 \approx L3 \approx 21.5$ nm. Sample bias $V = 100$ mV, tunneling current $I_t = 1.0$ nA. **c**, Representative dI/dV spectra taken in AA and AB regions of Supplementary Fig. 6a. The pseudo-Landau levels are indicated by numbers. **d**, Linear fit of the pseudo-Landau peaks and $\sqrt{N(N-1)}$ relations. The fitted PMF value is about 8 T.

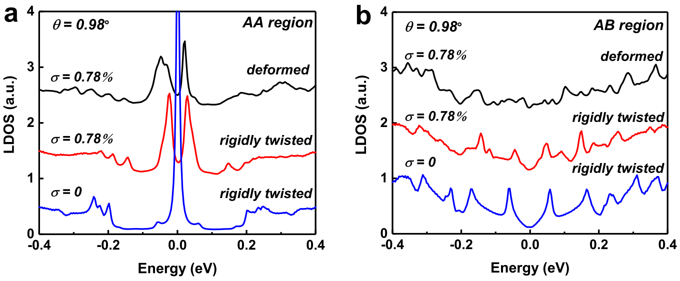

Supplementary Figure 8. **Electronic properties of TBG (twist angle $\theta = 0.98°$).** Local density of states for TBG with ($\sigma = 0.78\%$) and without heterostrain in **a** AA and **b** AB regions. Local density of states for deformed and rigidly TBG are also compared.

but exempt of heterostrain, strong localizations occurs only for zero energy in the AA region. Nevertheless, in other cases, two similar low peaks flank the Dirac point, which are attributed to the modification of the band structure by the heterostrain. Moreover, in the relaxed structure, well-defined Landau quantizations are

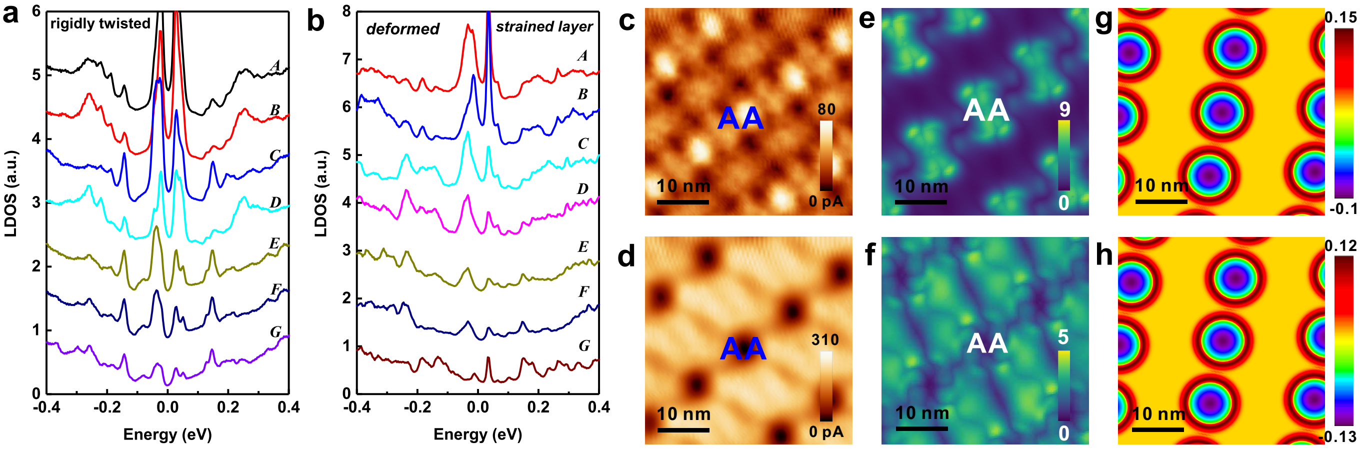

Supplementary Figure 9. **Electronic properties of TBG with twist angle $\theta = 0.98°$ and heterostrain $\sigma = 0.78\%$. a**, A series of calculated spectra of the layer without strain from the same positions in Fig.3**b** in the main text for the rigidly twisted case. **b**, Corresponding spectra of the strained layer with deformed case. **c**, Measured local density of states mapping with sample bias 184 mV and **d** with 110 mV. **e**, The calculated mapping with the energy 33 mV and **f** with the energy 98 mV. **g** and **h**, The calculated electrostatic potential V for the bottom and top layers, respectively.

induced in the high energy region and the low-energy peaks are broadened. Similar to what was reported in Ref. [2], high energy resonant peaks appear in the AB region in all cases, as shown in Supplementary Fig. 8b. These peaks, associated with a partial band gap opening in high energy moiré band, have minor changes in the presence of heterostrain and atomic deformation. This is expected since these lattice distortions only occur around the AA region.

Supplementary Note 9. The electronic properties of TBG with twist angle $\theta = 0.98°$ and heterostrain $\sigma = 0.78\%$

In this section, we present the electronic properties of TBG plotted in Fig. 4 in the main text. To verify that the generated non-uniform pseudo-magnetic field leads to pseudo-Landau levels, we also calculate the corresponding local density of states of the layer without strain for the rigidly twisted bilayer graphene with $\theta = 0.98°$ and $\sigma = 0.78\%$, as shown in Supplementary Fig. 9a. The appearances of the high energy resonances are dramatically different, especially in positions located close to the AA point. In Fig. 9b, we calculated the corresponding local density of states of the layer with strain in the deformed case and compare it with the results in Fig. 4 in the main text. Two low-energy peaks are observed, which are similar to that of the other layer without strain (see Fig. 4 in the main text). However, the high energy peaks are completely different from both the measured and calculated LDOS of the other layer without strain. That is, there are no such "pseudo-Landau peaks" in the local density of states even if the strained layer has the same structural deformation as the other one without strain. Supplementary Figure 9c and 9d shows the energy dependence of spatial distributions of dI/dV mappings. Here, in Supplementary Fig. 9c we can see that close to the first peak in the AA region (184 mV), the intensity of LDOS in AA regions is much higher than that in AB with distinct contrast, whereas in Supplementary Fig. 9d the intensity reverses when the measured energy approaches to the peak in AA region in the dI/dV curve (110 mV). Such strong local density of states modulation shows spatially localized electronic states with specific energies, resembling the charge density wave found in low-angle TBG [11]. The real-space distribution of eigenstates can be compared with the measurements of STM at particular energies. We utilize the Tight-Binding Propagation Method (TBPM) to calculate the so-called quasieigenstates, which are close to the real eigenstates [12]. In Supplementary Fig. 9e and 9f, clear moiré patterns can be found. Besides, localized states are clearly seen inside the AA regions, which are consistent with the dI/dV mappings observed in experiments. The local deformation potential V is also calculted by using the formula in Eq. 2 in the main text. Similar to the result of the TBG with $\theta = 0.48°$, the local deformation potential is periodic and non-uniform. Obviously, the V in the bottom layer is 0.035 eV higher than that of the top layer, which is due to the unaxial strain on the bottom layer.

Supplementary Note 10. The effect of the homostrain

We have investigated the effect of homostrain on the electronic properties of TBG with twist angle $\theta = 0.98°$. For comparison between homo and heterostrain, we per-

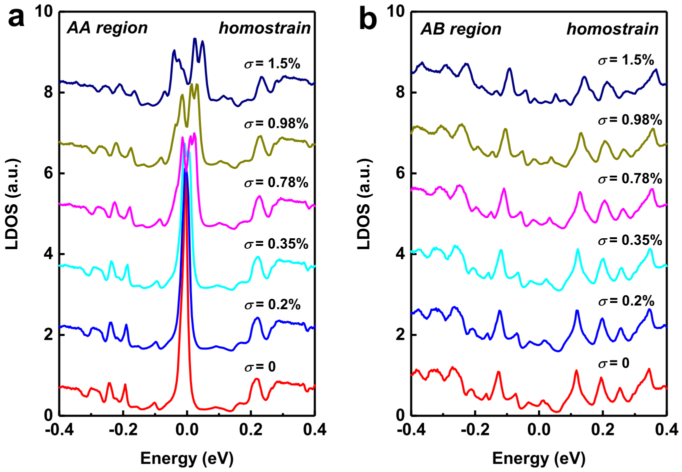

Supplementary Figure 10. **Calculated local density of states with homostrain.** Calculated local density of states in **a** AA region and **b** AB region for the twisted bilayer graphene with twist angle $\theta = 0.98^{\circ}$ and different uniaxial homostrain along the armchair chain direction of graphene. All curves have been shifted vertically for clarity.

formed tight-binding calculations with different uniaxial homostrain values along the armchair direction. Following Ref. [10], we constructed the system using the method in Supplementary Note 6. The calculated results in the AA and AB regions of TBG with various values for homostrain are shown in Supplementary Fig. 10. The local density of states in the AB region are similar to the situation without homostrain, which is only weakly affected even with a much larger value ($\sigma = 1.5\%$). On the contrary, the homostrain has a significant effect on the electronic properties in the AA region, especially at the low energies. For homostrains larger than $\sigma = 0.35\%$, the single low energy peak splits into two or three peaks. When the homostrain varies between values from $\sigma = 0\%$ to $\sigma = 1.5\%$, in the AA region, the high energy peaks have negligible changes, and the calculated LDOS are completely different from the measured ones in Fig. 4 in the main text. Moreover, the homostrain changes the moiré wavelengths in the real space by the same percentage along the homostrain direction. To obtain a moiré pattern similar to the one in Fig. 4a in the main text, the value of homostrain should be larger than 50%, which is unreasonable in a realistic sample. As a consequence, we exclude the homostrain as one source of strain in the BTG shown in Fig. 4 in the main text, and determine heterostrain as the origin of the observations.

* These two authors contributed equally
† s.yuan@whu.edu.cn
‡ syqin@ustc.edu.cn

Supplementary References

[1] I. Brihuega, P. Mallet, H. González-Herrero, G. Trambly de Laissardière, M.M. Ugeda, L. Magaud, J.M. Gómez-Rodríguez, F. Ynduráin, and J.-Y. Veuillen, Phys. Rev. Lett. **109**, 196802(2012).
[2] L. Huder, A. Artaud, T.L. Quang, G. Trambly de Laissardière, A.G.M. Jansen, G. Lapertot, C. Chapelier and V.T. Renard, Phys. Rev. Lett. **120**, 156406 (2018).
[3] Li. Gao, Guest. Jeffrey, Guisinger. Nathan P, Nano Letters **10**, 3512-3516 (2010).
[4] Matthias Batzill, Surface Science Reports **67**, 83–115 (2012).
[5] Wei Chen, Hua Chen, Haiping Lan, Ping Cui, Tim P. Schulze, Wenguang Zhu, and Zhenyu Zhang, Phys. Rev. Lett. **109**, 26507 (2012).
[6] A. Artaud, L. Magaud, T. Le Quang, V. Guisset, P. David, C. Chapelier and J. Coraux, Sci. Rep. **6**, 25670 (2016).
[7] K. Hermann, J. Phys.: Condens. Matter **24**, 314210 (2012).
[8] M.M. van Wijk, A. Schuring, M.I. Katsnelson and A. Fasolino, 2D Mater. **2**, 034010 (2015).
[9] F. Gargiulo adn O. Yazyev, 2D Mater. **5**, 015019 (2018).
[10] V. Hung Nguyen and P. Dollfus, 2D Mater. **2**, 035005 (2015).
[11] G. Li, A. Luican, J.M.B. Lopes dos Santos, A.H. Castro Neto, A. Reina, J. Kong and E.Y. Andrei, Nat. Phys. **6**, 109-113 (2010).
[12] S. Yuan, H. De Raedt and M.I. Katsnelson, Phys. Rev. B **82**, 115448 (2010).
